# Length–weight relationships and condition factors of mullets *Liza macrolepis* and *Moolgarda engeli* (Pisces: Mugilidae) harvested from Lambada Lhok waters in Aceh Besar, Indonesia

**DOI:** 10.12688/f1000research.22562.2

**Published:** 2020-06-10

**Authors:** Derita Yulianto, Indra Indra, Agung Setia Batubara, Deni Efizon, Firman M. Nur, Syamsul Rizal, Roza Elvyra, Zainal Abidin Muchlisin

**Affiliations:** 1 Doctorate Program in Mathematics and Applied Sciences,, Universitas Syiah Kuala, Banda Aceh, Aceh province, 23111, Indonesia; 2 Faculty of Agriculture, Universitas Syiah Kuala, Banda Aceh, Aceh province, 23111, Indonesia; 3 Faculty of Marine and Fisheries, Universitas Syiah Kuala, Banda Aceh, Aceh Province, 23111, Indonesia; 4 Marine and Fisheries Research Center, Universitas Syiah Kuala, Banda Aceh, Aceh Province, 23111, Indonesia; 5 Faculty of Fisheries and Marine Sciences, Universitas Riau, Pekanbaru, Riau Province, Indonesia; 6 Faculty of Mathematics and Natural Sciences, Universitas Riau, Pekanbaru, Riau Province, Indonesia

**Keywords:** Growth pattern, Relative weight condition factor, Fulton’s condition factor, Linear allometric model

## Abstract

**Background:** The mullets fish *Liza macrolepis* and *Moolgarda engeli* are predominant in the Lambada Lhok waters in Aceh province. At present, no scientific report on this species in Aceh waters is available. Therefore, the objective of the present study is to examine the growth pattern and condition factor of the species of mullets *L. macrolepis *and *M. engeli* harvested from the aforementioned coastal waters.

**Methods:** The sampling was done in three locations in the Lambada Lhok waters from July to November 2018. The fish were captured using gillnets from 6:00 AM to 3:00 PM four times a month for five months.  A total of 242 *L. macrolepis* and 109 *M. engeli* were used for the analysis. The growth pattern was analyzed using linear allometric model; then, two condition factors, Fulton’s and relative weight, were calculated.

**Results:** The study revealed a ***b*** value of 2.49 for the male *L. macrolepis* and 1.81 for the female. The ***b*** value was 3.22 for the male *M. engeli* and 3.41 for the female. The ***b*** value of the fish was higher during the dry season. The Fulton’s condition factor of the male *L. macrolepis *was 1.19, and that of the female was 1.19. The relative condition factor of this species was 100.11 and 100.01 for males and females, respectively. The Fulton condition factor of male *M. engeli* was 1.05 and that of the female was 1.06. The relative weight condition factors were 101.08 and 100.61 for the male and female, respectively.

**Conclusions:** The growth pattern of *M. engeli* tends to be isometric, whereas that of *L. macrolepis* has a negative allometric growth pattern. The condition factors indicate that the Lambada Lhok waters are still in good condition and support the growth of the mullets, but *M. engeli* is more adaptable than *L. macrolepis*.

## Introduction

Mullets (Mugilidae) represent a family of euryhaline fish that can tolerate a wide range of salinity ^1, 2^. This fish is frequently found in marine environments, brackish and fresh water ^2– 7^. To date, a total of 30 genus belonging to 78 species of mullets have been described worldwide ^8, 9^. A total of 21 species of mullets have been reported in Indonesian waters, and among them, four species have been recorded in the waters of Aceh province; these are *Liza melinoptera, Mugil chepalus, Valamugil cunnesius,* and *V. speigleri* ^4, 10, 11^. Our previous study recorded three additional mullets from Aceh waters, namely, *L. macrolepis, Crenimugil crenilapis,* and *Moolgarda engeli* (Yulianto, thesis in preparation), which accounted for a total of seven species of mullets in the Aceh region. These additional species are commonly found in Lambada Lhok, Aceh Besar district close to Banda Aceh City, the capital of Aceh province, Indonesia. Moreover, our field observation showed that *L. macrolepis* and *M. engeli* were the predominant species among other mullets in this area.

The coastal area of Lambada Lhok has a mangrove forest; however, the forest area has been significantly decreased due to tsunami disaster in 2004, land conversion for settlement and aquaculture ponds. The other potential threats are pollution from domestic waste, fishing port, and tourism activities ^12, 13^. Mullets are a species of shoaling and schooling fish commonly found in river mouths for feeding ^14, 15^, which then subsequently migrate to deep waters for spawning ^16^. Therefore, these fish are highly susceptible to exposure to pollution from coastal areas; for instance, *Chelon subviridis* from Donan River estuary, Central Java, have been contaminated by cadmium and copper ^17^. A similar finding has been reported in *M. cephalus* from the Ligurian Sea in Italy ^18^. In addition, *L. macrolepis* and *M. engeli* have been harvested intensively by local fishermen, thereby increasing the pressure on these fish. Thus, research related to bioecology as basic information is crucial in planning an effective conservation strategy. The two important pieces of information are length–weight relationships (LWRs) and growth pattern and condition factors.

The study of the LWRs and condition factors has become popular and is therefore commonly conducted by fish biologists ^19^. The objectives of LWRs study are to determine the specific weight and length variations of fish individually or the population a whole to determine the age, obesity status, health, productivity, and physiological conditions, including gonadal development ^20, 21^. LWRs analysis is also useful to estimate the fish condition or plumpness index, which is an important variable in the evaluation of the health conditions of fish populations or individuals ^22– 24^. The condition factor indicates the biological and physical conditions of fish and its fluctuations by interaction among feeding condition, food reserves, and parasite infestation ^25– 27^.

Several studies on LWRs and condition factor of mullets have been conducted, such as, that on *M. dussumieri* in Ujung Pangkah, East Jawa, Indonesia ^14^; on LWRs in *L. macrolepis* from Indian waters ^28, 29^, and on *L. macrolepis* from Taiwanese waters ^30^. In addition, the study of LWRs in several species of mullets in Aceh waters, Indonesia has been reported by Mulfizar *et al.* ^31^ in *M. chepalus* from the waters of Kuala Gigieng, Aceh Besar, and by Muttaqin *et al.* ^32^ in the same species from Madat waters in East Aceh. However, no study has been published on *L. macrolepis* and *M. engeli*. Thus, the objectives of the present study are to analyze LWRs and condition factor of these two types of mullet harvested from Lambada Lhok waters.

## Methods

### Time, site, and sampling

**Figure 1.**
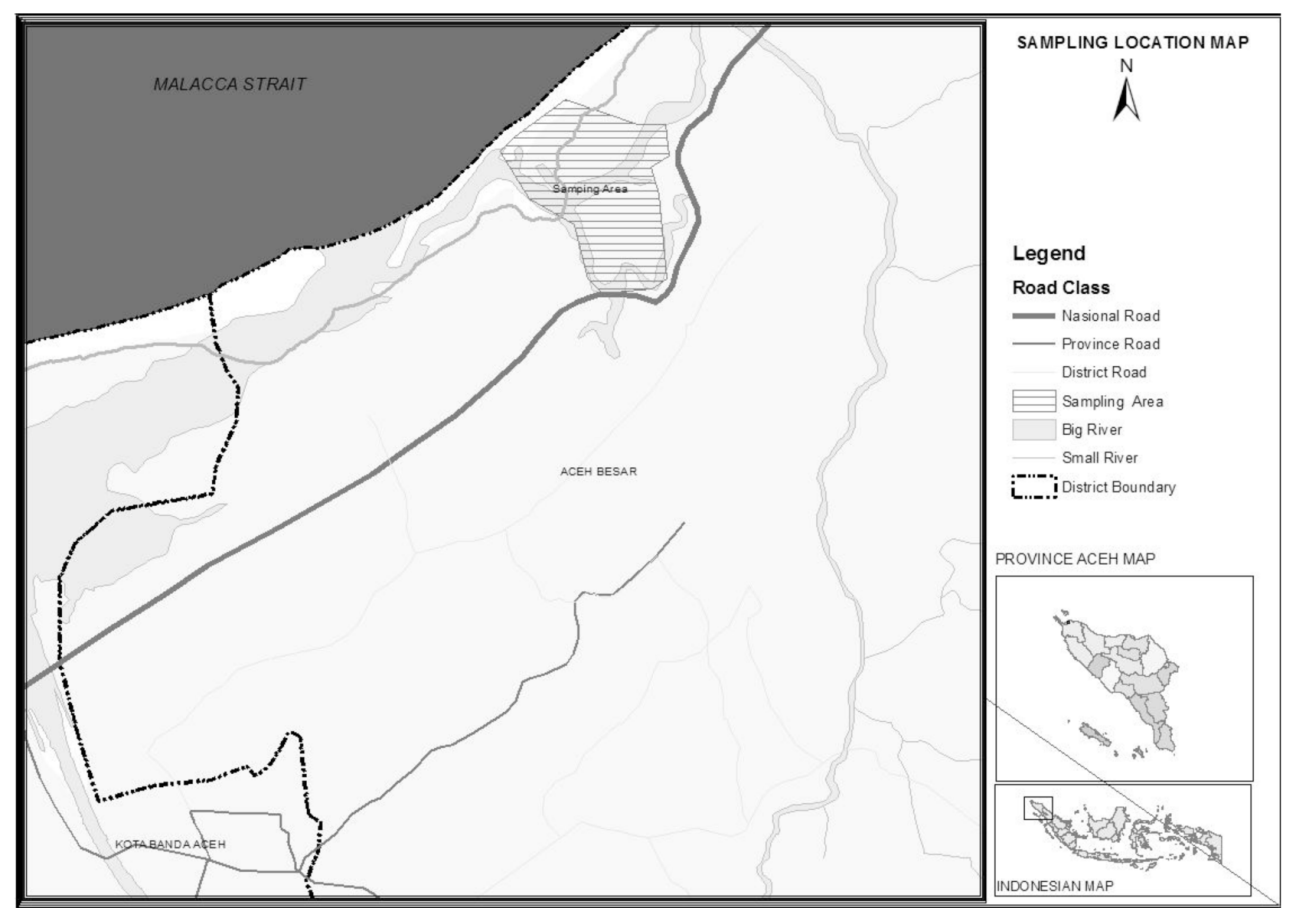
Map of Lambada Lhok coastal area showing the sampling location (5 ° 36'57.6 "N, 95 ° 23 '25.6" E).

The sampling was conducted from July to November 2018 in the Lambada Lhok estuary, Aceh Besar regency in Aceh province, Indonesia ( Figure 1). Sampling times represent the dry season (July - September) and the wet season (October - December). The location is a small river mouth and deforested mangrove areas and fishing port (5°36'57.6" N, 95° 23'25.6" E). The fish sampling was done purposively at these locations as they were easy to access and fish were reported to be present. Sampling was done purposively at these locations as they were easy to access and fish were reported to be present. Sampling was conducted four times a month for five months. The target species in this study were determined based on preliminary survey and observations on the composition of local fishermen catches, where *Liza macrolepis* and *Moolgarda engeli* were the dominant species caught.

The fish was caught using gillnets with a mesh size of 2.0 inch. The gillnets were set up in the waters for 4 h (06.00 AM –03.00 PM) and were monitored in 30 min intervals. The sampled fish was washed, euthanized with cold water 4 °C for 5 min. This euthanize method was chosen as it is easy to apply, non-toxic and inexpensive. Then the fish was preserved temporarily in crushed ice in a styrofoam box, and then transported to a laboratory for further analysis. Other species of fish caught were separated from the mullets, and released back to the waters if still live, but the fish taken for consumption if they died during sampling. All efforts were made to ameliorate harm to the animals by complying to the guidelines of ethics animal use in research of Syiah Kuala University.

## Field observations

During this process, the weather condition, tides, and water turbidity are also observed visually during the sampling.

### LWRs analyses

The length-weight relationships were calculated to predict the growth pattern of the fish. A total of 242 *L. macrolepis* and 109 *M. engeli* were measured for total length to the nearest mm using a digital caliper (Mitutoyo CD-6CS, error: 0.05 mm) and for body weight to the nearest mg using a digital balance (ION EPS05, error: 0.1 mg). Male and female fish were calculated for LWRs separately. A linear allometric model (LAM) was used to calculate parameters ***a*** and ***b*** values based on the work of De-Robertis and Williams ^33^ and Muchlisin *et al.* ^22^ as follows:

$$\text{W}={\text{e}}^{0.5\sigma }.\text{a}.{\text{L}}^{\text{b}},$$

where W is total body (g), L is total length (mm), *a* is regression intercept, *b* is regression coefficient, σ is residual variation of the LAM, and *0.5* is correction factor. The growth pattern of the fish is divided into three categories; isometric when the *b* value is equal to 3, negative allometric when the *b* value lower than 3, and positive allometric when the *b* value is higher than 3.

### Condition factor analyses

The condition factor indicates the conditions of the fish and water, and their interactions. Two condition factors, namely, relative weight condition factor (Wr) and Fulton’s condition factor (K) were analyzed in this study. The relative weight condition factor of 100 indicates a balance between prey and predator, while if the Wr higher than 100 indicates a surplus of prey, and it vice versa. Based on the work of Rypel and Richter ^34^, the relative weight condition factor was calculated as follows: Wr = (W/Ws) × 100, where Wr is the relative weight condition factor, W is body weight of fish from direct measurement, Ws is the prediction weight of fish, and Ws = aL ^b^.

Based on the work of Okgerman ^35^, Fulton’s condition factor was calculated as follows:

$$\text{K}={\text{WL}}^{–3}\times 100,$$

where K is the Fulton’s condition factor, W is the body weight of fish from direct measurement (g), and L is the total length of fish from direct measurement (mm). According to Morton and Routledge ^36^, a fish population is in good condition when the K value is higher than 1.

### Data analysis

The raw data of total length and body weight were processed using a Microsoft Excel (Microsoft Office 365). The data were presented as tables and figures, and then the data were analyzed descriptively through comparison with related reports, theories, and field observations.

## Results

### Length-weight relationships

The field observation of the catch composition showed that *Liza macrolepis* and *Moolgarda engeli* were predominant. A total of 242 *L. macrolepis* and 109 *M. engeli* were sampled and measured in the study. The length of male *L. macrolepis* ranged from 141.4 – 202.1 mm (164.8 ± 15.03 mm), and ranged from 129.2–185.4 mm (159.1 ± 12.66 mm) for females. The body weight of male *L. macrolepis* ranged from 34.7 g to 89.6 g (54.1 ± 13.3 g in average), and 28.8 g to 75.13 g (47.9 ± 9.52 g in average) in females. The length of the male *M. engeli* ranged from 109.9–188.5 mm (161.9±20.83 mm) and 116.5–182.3 mm (154.1 ± 18.94 mm) for females. The body weight of the males was 13.6 – 108.5 gram (47.6±19.3 gram), and that of the females was 14.2–75.1 gram (41.4 ± 16.43 gram). Raw data are available as underlying data ^37^.

**Figure 2.**
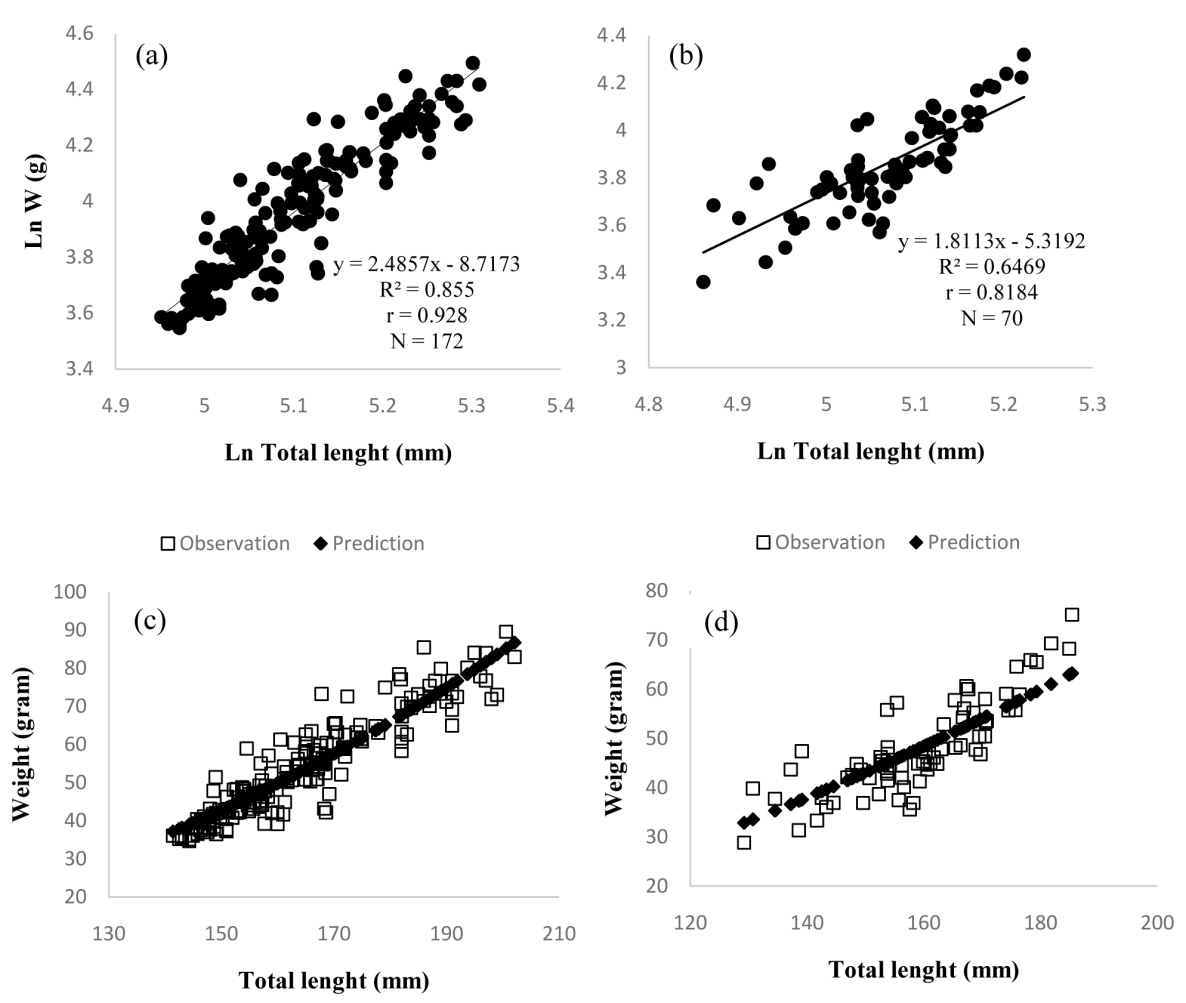
The length-weight relationship of *Liza macrolepis* based on linear allometric model ( **a**) male, ( **b**) female; Comparison of observed and predicted growth for male ( **c**), and female ( **d**) of *Liza macrolepis*. R2 - determination coefficient, r - correlation coefficient, N - number of fish sampled.

**Figure 3.**
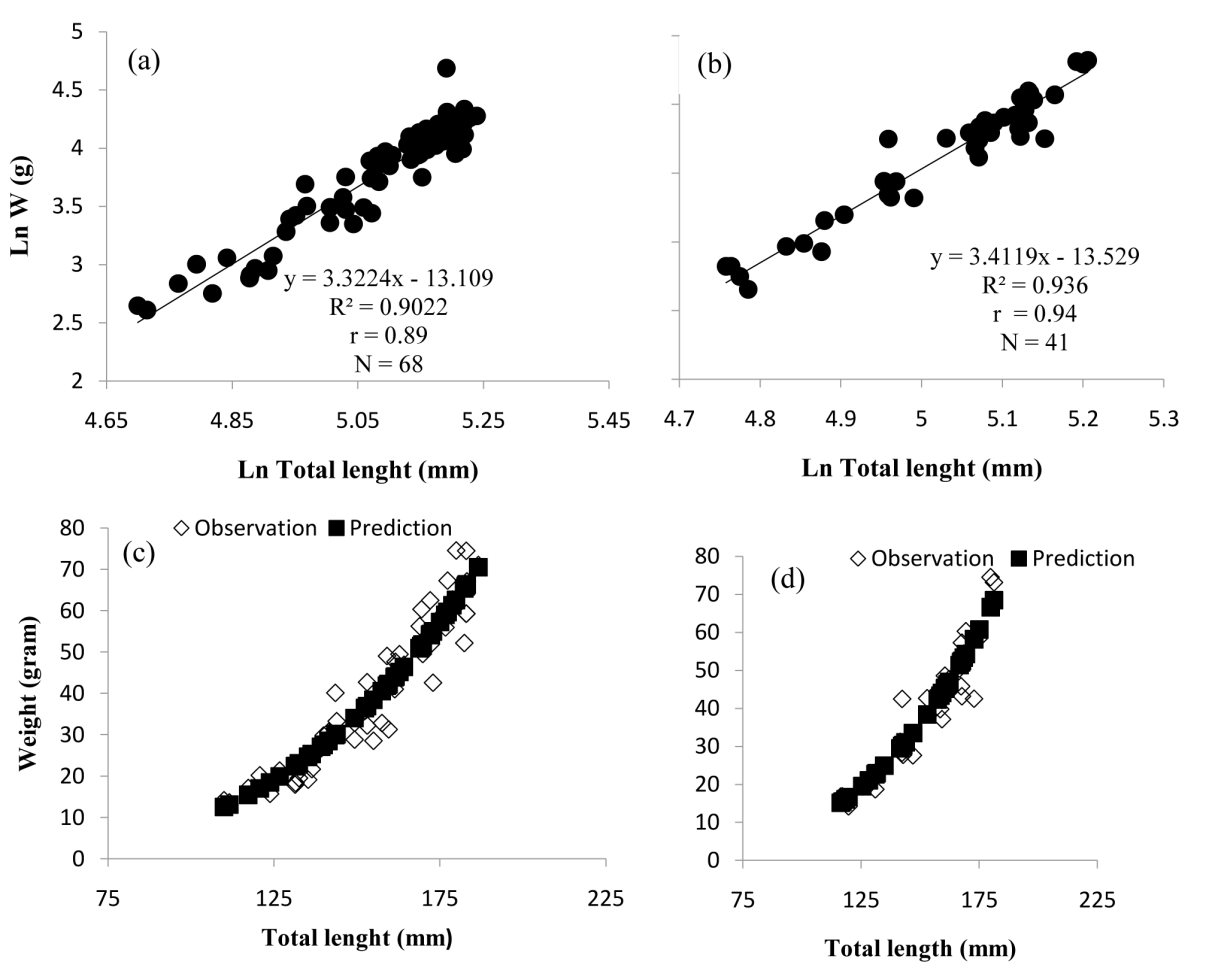
The length-weight relationship of *Moolgarda engeli* based on linear allometric model ( **a**) male, ( **b**) female; Comparison of observed and predicted growth for male ( **c**), and female ( **d**) of *Moolgarda engeli*. R2 - determination coefficient, r - correlation coefficient, N - number of fish sampled.

The results of the LWRs analysis on *L. macrolepis* showed that the male fish had a ***b*** value of 2.49 with a correlation coefficient of 0.93, and the female fish had a ***b*** value of 1.81 and a correlation coefficient of 0.82 ( Figure 2a and 2b). Therefore, the male and female *L. macrolepis* displayed negative growth patterns, and a moderate correlation between body weight and total length of the fish. The results of LWRs analysis of male *M. engeli* revealed that the average ***b*** value was 3.22 with a coefficient correlation of 0.89. The female *M. engeli* had an average ***b*** value of 3.41 with a coefficient correlation of 0.93 ( Figure 3a and 3b). These data indicate that the male and female *M. engeli* have a positive allometric growth pattern, and a strong correlation between body weight and total length.

**Figure 4.**
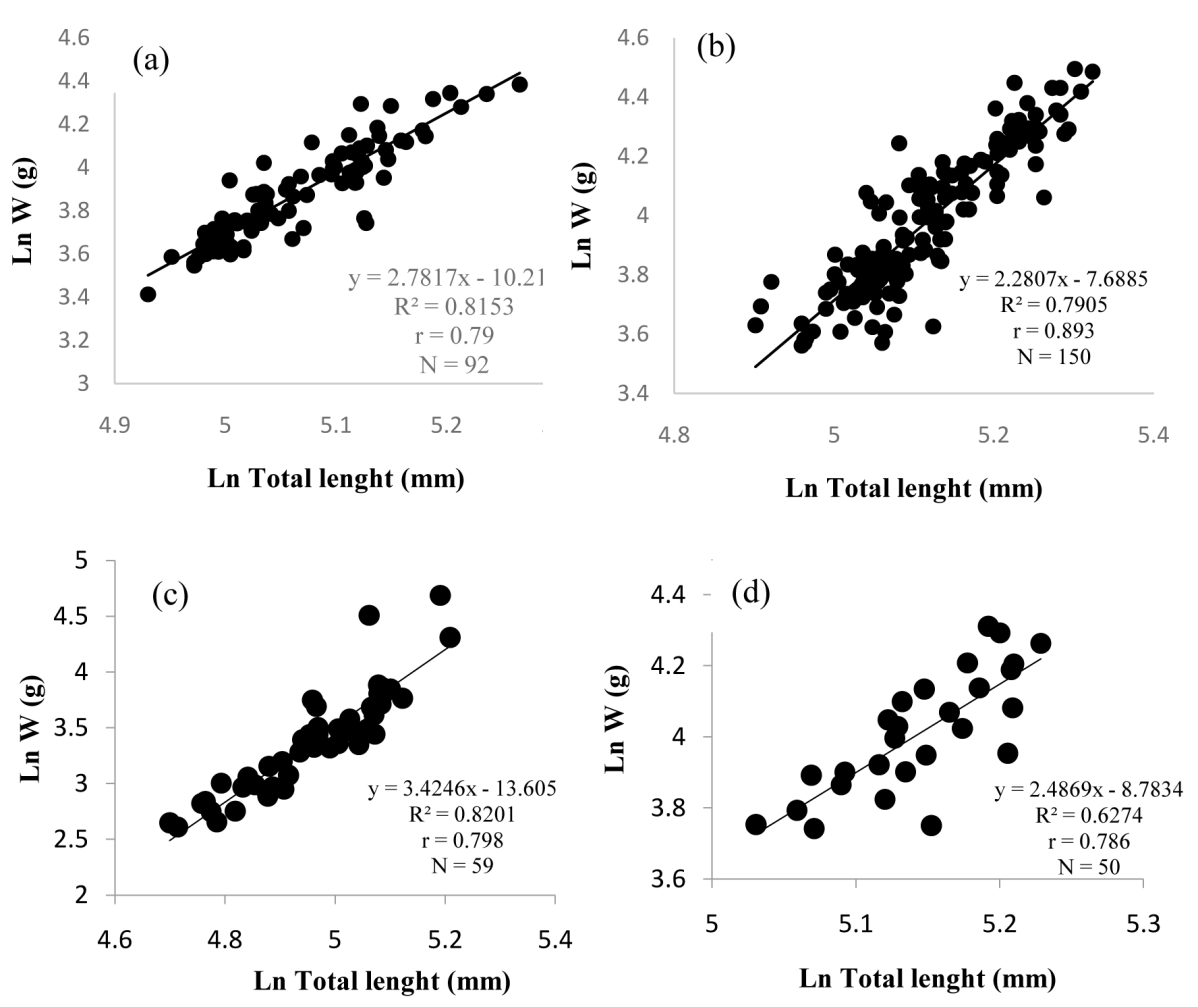
The length-weight relationship of *Liza macrolepis* ( **a**) during dry season ( **b**) during wet season and *Moolgarda engeli* ( **c**) during dry season ( **d**) during wet season. R2 - determination coefficient, r - correlation coefficient, N - number of fish sampled.

Based on sampling season, the average ***b*** value of *L. macrolepis* (male and female) was 2.78 during the dry season and 2.28 during the wet season ( Figure 4a). The ***b*** value of *M. engeli* was 3.42 during the dry season and 2.48 during the wet season ( Figure 4b). These data indicate that the ***b*** value is lower during the wet season for both species. The scatter plots of predicted standard weight for respective observed length, as calculated from the composite of length–weight regression, are presented in Figure 2c and 2d and Figure 3c and d. The regression models show a difference between the observed and predicted growth patterns in both species.

***Condition factors***. The results showed that the male *L. macrolepis* had a Fulton’s condition (K) factor of 1.19, and relative weight condition factor (Wr) of 100.11; while the females had a Fulton’s condition factor of 1.19, and relative weight condition factor of 100.01. In addition, male *M. engeli* had a Fulton’s condition factor of 1.05, and relative weight condition factor of 101.08; whereas the females has Fulton’s condition factor of 1.06, and relative weight condition factor of 100.61 ( Table 1). Based on sampling season, the Fulton’s condition of *L. macrolepis* during the dry season was 1.22 and 100.49 for the relative weight; during the wet season these were 1.19 and 101.74, respectively. In addition, the K and Wr values of *M. engeli* during the dry season were 1.03 and 102.09, respectively; during the wet season these were 1.09 and 100.47, respectively ( Table 2). The data of the weather condition were observed visually. The days are mostly rainy during sampling in the wet season, and therefore the turbidity was higher during this season.

**Table 1.** The b value, coefficient of correlation and determination, and condition factors of *Liza macrolepis* dan *Moolgarda engeli* sampled from July-November 2018 according to sex.

Species	Sex	Body weight (mean ±SD) (gram)	Total length (mean ± SD) (mm)	N	*b* value	Coefficient of correlation (r)	Coefficient of determination (R ^2^)	Fulton condition factor (K)	Relative weight condition factor
									(Wr)
*L. macrolepis*	Male	34.7 – 89.6 (54.1 ± 13.3)	141.4 - 202.1 (164.8 ± 15.03)	172	2.49	0.93	0.85	1.19 ± 0.12	100.11 ± 09.38
	Female	28.8 – 75.1 (47.9 ± 9.52)	129.2 – 185.4 (159.1 ± 12.66)	70	1.81	0.82	0.65	1.19 ± 0.19	100.01 ± 11.23
*M. engeli*	Male	13.6 – 108.5 (47.6 ± 19.3)	109.9 – 188.5 (161.9 ± 20.83)	68	3,22	0.89	0.90	1.05 ± 0.15	101.08 ± 14.74
	Female	14.2 – 75.1 (41.4 ± 16.43)	116.5 – 182.3 (154.1 ± 18.94)	41	3.41	0.93	0.94	1.06 ± 0.25	100.61 ± 11.72

**Table 2.** The b value, coefficient of correlation and determination, and condition factors of *Liza macrolepis* dan *Moolgarda engeli* sampled from July-November 2018 according to sampling seasons.

Species	Season	Body weight (mean ±SD) (gram)	Total length (mean ± SD) (mm)	N	*b* value	Coefficient of correlation (r)	Coefficient of determination (R ^2^)	Fulton condition factor (K)	Relative weight condition factor
									(Wr)
*L. macrolepis*	Dry	30.4 – 80.2 (49.6 ± 11.17)	138.4 - 193.7 (158.9 ± 11.37)	92	2.78	0.90	0.82	1.22 ± 0.11	100.49 ± 59.56
	Wet	34.7 – 85.5 (53.9 ± 13.35)	129.2 – 202.1 (159.1 ± 12.66)	150	2.28	0.89	0.79	1.19 ± 0.15	100.58 ± 10.89
*M. engeli*	Dry	13.6 – 108.5 (32.1 ± 18.42)	109.9 – 182.9 (142.4 ± 17.51)	59	3.42	0.79	0.82	1.03 ± 0.29	102.09 ± 24.18
	Wet	41.2 – 74.5 (56.2 ± 9.31)	153.1 – 188.5 (172.1 ± 8.97)	50	2.48	0.78	0.63	1.09 ± 0.11	100.47 ± 09.81

## Discussion

The study revealed that male and female *L. macrolepis* had negative allometric growth patterns. However, the ***b*** value of the females was less than that of the male. The ***b*** value of the male *M. engeli* showed a positive allometric growth pattern. Based on these growth pattern data, the study indicated that *M. engeli* grows better than *L. macrolepis*, thereby indicating that *M. engeli* is more adaptable to the environmental condition of Lambada Lhok waters. Furthermore, the field observation on the catch composition of the fishermen showed that *M. engeli* was also predominant. The Fulton’s condition factor showed a slight difference in K value between the male and female for both species, where the K value was higher than 1. According to Morton and Routledge ^36^, a fish population is in good condition when the K value is higher than 1. The study showed that the K value of *L. macrolepis* ranged from 1.16 to 1.22, and 1.03 to 1.09 for *M. engeli*; therefore, both populations are in good condition, in dry and wet season, respectively. In addition, the relative weight condition factor of both species is close to 100, indicating a balance between prey and predator ^20^. These results show that these waters provide a sufficient food source for these species. The relative weight condition factor also corresponds to fish health conditions, stock estimates, and management levels ^23, 35, 36, 38, 39^. Therefore, the Lambada Lhok waters provide sufficient food sources for mullets.

The results also showed differences in growth patterns during the dry and wet seasons, and that the fish grew better during the dry season. The probable reason is that the waters are clear and a maximum rate of sunlight penetrates into the waters, triggering the growth of phytoplankton and algae. Algae is a primary food item for the mullets ^40– 43^. By contrast, turbidity and currents were higher during the wet season ^44^, and thereby presume to inhibit the growth of phytoplankton and algae as important food item for mullets ^42, 45^. A similar phenomenon was reported by Chu *et al.* ^30^, who found a negative growth pattern in *L. macrolepis* in Taiwan during winter, and an isometric growth pattern during summer and spring. Moreover, Sandhya and Shameem ^29^ observed a negative growth pattern in *L. macrolepis* in polluted waters, in contrast to an isometric growth pattern in unpolluted waters. However, a contrary finding was reported in five species of fish ( *Barbus intermedius, Clarias gariepinus, Labeo cylindricus, Oreochromis niloticus baringoensis* and *Protopterus aethiopicu*) in the Lake Baringo, Kenya ^46^ where these species are growing well during wet season ^46^. Therefore, they concluded that season affected significantly on the LWRs, but did not affect the condition factor of fish ^46^. A negative growth pattern was also reported in three species of mullets ( *Parachelon grandisquamis, Neochelon falcipinnis* and *Mugil cephalus*) in the Sombreior River, Niger Delta, Nigeria ^47^. According to Blackwell *et al*. ^20^ the Wr is useful to estimate fish health conditions, stock, and management levels of fisheries resources.

According Muchlisin *et al.* ^22^, besides being affected by the environmental factors, the growth pattern of fish is also influenced by fish behavior; for example, the fish that were active swimmer had a lower ***b*** value than those that were passive swimmers ^48– 50^.

The average correlation coefficients of *L. macrolepis* were 0.93 and 0.89 in females and males, respectively, whereas *M. engeli* had a correlation coefficient of 0.89 for males and 0.93 for females. In general, the correlation coefficients of *L. macrolepis* and *M. engeli* tend to be similar (above 75%), indicating a strong correlation between total length and body weight. The determination coefficients of *L. macrolepis* were 0.85 and 0.65 for the male and female fish, respectively, which means that approximately 65%–85% of total variants can be explained by the model, while *M. engeli* had a value of 0.90 and 0.94 for males and females, respectively, indicating that 90%–94% of variants can be explained by the model.

## Conclusion

The results of this study showed that *L. macrolepis* had a negative growth pattern, whereas *M. engeli* had a positive allometric growth pattern. These growth patterns were better during the dry season for both species. The Fulton’s condition factor of the male *L. macrolepis* and *M. engeli* were higher than 1 and the relative weight condition factors of both species tend to 100, indicate the environmental condition of Lambada Lhok remains suitable for the growth of the mullets, and the density of prey and predator is balanced.

## Data availability

### Underlying data

Figshare: Raw Data of *Liza macrolepis*.xlsx. https://doi.org/10.6084/m9.figshare.12028062.v1 ^37^

This project contains the following underlying data:

- Raw Data of *Liza macrolepis*.xlsx (Raw data of sampled *Liza macrolepis*)
- Raw Data of *Moolgarda engeli*.xlsx (Raw data of *Moolgarda engeli*)

Data are available under the terms of the Creative Commons Attribution 4.0 International license (CC-BY 4.0).
