# Supplementary material for: Length–weight relationships and condition factors of mullets  Liza macrolepis and  Moolgarda engeli (Pisces: Mugilidae) harvested from Lambada Lhok waters in Aceh Besar, Indonesia
Source: F1000Res. 2020 Jun 10;9:259. Originally published 2020 Apr 14. [Version 2] doi: 10.12688/f1000research.22562.2 (PMC7324949; doi:10.12688/f1000research.22562.2)
Supplement: Peer review history

# Peer review: Reviewer response for version 2

**DOI:** 10.5256/f1000research.27202.r64535

**Published:** 2020-06-29

**Authors:** Esti Handayani Hardi

**Affiliations:** 1 Animal physiology, development and molecular laboratory, Department of Biology, Faculty of Mathematics and Natural Sciences, Mulawarman University, Samarinda, Indonesia

I am in agreement with the revised manuscript and the article has improved significantly in method and discussion. The corrections made by the author(s) are fully satisfactory and based on my expertise, I now recommend this paper be approved by F1000Research.

Is the work clearly and accurately presented and does it cite the current literature?

Partly

If applicable, is the statistical analysis and its interpretation appropriate?

Yes

Are all the source data underlying the results available to ensure full reproducibility?

No source data required

Is the study design appropriate and is the work technically sound?

Yes

Are the conclusions drawn adequately supported by the results?

Partly

Are sufficient details of methods and analysis provided to allow replication by others?

Partly

Reviewer Expertise:

NA

I confirm that I have read this submission and believe that I have an appropriate level of expertise to confirm that it is of an acceptable scientific standard.

# Peer review: Reviewer response for version 2

**DOI:** 10.5256/f1000research.27202.r64534

**Published:** 2020-06-15

**Authors:** Rudy Agung Nugroho

**Affiliations:** 1 Animal Physiology, Development, and Molecular Laboratory, Department of Biology, Faculty of Mathematics and Natural Sciences, Mulawarman University, Samarinda, Indonesia

I am glad to note that the manuscript has now improved significantly and is better than the previous submission. The corrections made by the author(s) are fully satisfactory and I can judge, based on my expertise, that now I recommend this paper can be approved and indexed

Is the work clearly and accurately presented and does it cite the current literature?

Yes

If applicable, is the statistical analysis and its interpretation appropriate?

Yes

Are all the source data underlying the results available to ensure full reproducibility?

Yes

Is the study design appropriate and is the work technically sound?

Yes

Are the conclusions drawn adequately supported by the results?

Partly

Are sufficient details of methods and analysis provided to allow replication by others?

Partly

Reviewer Expertise:

Animal Physiology, Fish nutrition, Fish Conservation

I confirm that I have read this submission and believe that I have an appropriate level of expertise to confirm that it is of an acceptable scientific standard.

# Peer review: Reviewer response for version 1

**DOI:** 10.5256/f1000research.24904.r63617

**Published:** 2020-06-05

**Authors:** Rudy Agung Nugroho

**Affiliations:** 1 Animal Physiology, Development, and Molecular Laboratory, Department of Biology, Faculty of Mathematics and Natural Sciences, Mulawarman University, Samarinda, Indonesia

**Title:**

 The title is sufficient and reflects the content of the study.

**Abstract:**

 Well-constructed.

**Introduction:**

 Adequate.

**Materials and Methods:**

1. Please mention in the Time, site, and sampling or LWRs analyses section that male and female fish were measured/analysis for LWR separately.
2. In the fields observation, it is stated that, the weather condition, tides, and water turbidity are also observed visually during the sampling. Is the observed visual data recorded? If yes, please mention in the result section.

 

**Results:**

1. Please add field observation results (if the data was recorded as mentioned in the Materials and methods).
2. In the Condition factors section, it mentions about the dry session and rainy session; which dry or rainy session between July and November? Please add information.

 

**Discussion:**

1. Develop the discussion by mentioning about the relationship between the environment factor and the results of LWRs and CF fishes', ex: what prey and predator of both fish.
2. It is also mentioned in the discussion that the relative weight condition factor also corresponds to fish health conditions, stock estimates, and management levels. This can be an interesting discussion to be developed.

**Conclusion:**

 Sufficient, but it is better to add a conclusion regarding the condition factors related to the sex (male/female) of both fish.

**References:**

 Sufficient.

Is the work clearly and accurately presented and does it cite the current literature?

Yes

If applicable, is the statistical analysis and its interpretation appropriate?

Yes

Are all the source data underlying the results available to ensure full reproducibility?

Yes

Is the study design appropriate and is the work technically sound?

Yes

Are the conclusions drawn adequately supported by the results?

Partly

Are sufficient details of methods and analysis provided to allow replication by others?

Partly

Reviewer Expertise:

Animal Physiology, Fish nutrition, Fish Conservation

I confirm that I have read this submission and believe that I have an appropriate level of expertise to confirm that it is of an acceptable scientific standard, however I have significant reservations, as outlined above.

# Response

**Published:** 2020-06-06

**Authors:** Zainal Abidin Muchlisin

**Affiliations:** Syiah Kuala University, Indonesia

1. Comment:

 Please mention in the Time, site, and sampling or LWRs analyses section that male and female fish were measured/analyzed for LWR separately.

 Respond:

 We have added this information in the material and method (page 5)

 2. Comment:

 In the field observation, it is stated that the weather condition, tides, and water turbidity are also observed visually during the sampling. Is the observed visual data recorded? If yes, please mention in the result section.

 Respond:

 This information was added in the results section (condition factor subsection) on page 8

 4. Comments:

 In the condition factors section, it mentions about the dry session and rainy session; which dry or rainy session between July and November? Please add the information.

 Respond:

 This detail description of the dry and wet/rainy seasons have been added at Time, Site and Sampling Section (page 4)

 5. Comment:

 Develop the discussion by mentioning the relationship between the environment factor and the results of LWRs and CF fishes', ex: what prey and predator of both fish.

 Respond:

 We have discussed the relationship between environmental factors and LWRs and the condition factor of the fish (page 10-11). Based on some references, and our data (these data will be published separately) that the prey (food) for the mullets are mostly plankton and algae (we have added this information in the discussion section). But, we have not examined the predator for mullets.

 6. Comment:

 It is also mentioned in the discussion that the relative weight condition factor also corresponds to fish health conditions, stock estimates, and management levels. This can be an interesting discussion to be developed.

 Respond:

 We have developed the discussion as suggested by the reviewer (page 11 under quotation to Blackwell et al. (2000).

 7. Comment:

 The conclusion is sufficient, but it is better to add a conclusion regarding the condition factors related to the sex (male/female) of both fish. 

 Respond:

 We have extended the conclusion, but we think that the detailed information male and female fish would be better displayed in the Abstract. In the conclusion section, we mentioned generally.

# Peer review: Reviewer response for version 1

**DOI:** 10.5256/f1000research.24904.r63616

**Published:** 2020-06-01

**Authors:** Esti Handayani Hardi

**Affiliations:** 1 Animal physiology, development and molecular laboratory, Department of Biology, Faculty of Mathematics and Natural Sciences, Mulawarman University, Samarinda, Indonesia

- The method of taking fish has not been clearly described in the Methods.
- The water quality or environment condition has not been mentioned.
- Maybe, add the discussion about the relationship between the environment with the fishes' conditions.
- Many references were old references, need to add the new articles.
- The conclusion part is not enough.

Is the work clearly and accurately presented and does it cite the current literature?

Partly

If applicable, is the statistical analysis and its interpretation appropriate?

Yes

Are all the source data underlying the results available to ensure full reproducibility?

No source data required

Is the study design appropriate and is the work technically sound?

Yes

Are the conclusions drawn adequately supported by the results?

Partly

Are sufficient details of methods and analysis provided to allow replication by others?

Partly

Reviewer Expertise:

fish diseases, microbiology, aquaculture biotechnology

I confirm that I have read this submission and believe that I have an appropriate level of expertise to confirm that it is of an acceptable scientific standard, however I have significant reservations, as outlined above.

# Response

**Published:** 2020-06-06

**Authors:** Zainal Abidin Muchlisin

**Affiliations:** Syiah Kuala University, Indonesia

1. Comment:

The method of taking fish has not been clearly described in the Methods.

Respond:

We have mentioned that  “The fish sampling was done purposively at these locations as they were easy to access and fish were reported to be present” (page 4)

2. Comment:

The water quality or environment condition has not been mentioned

Respond:

We have not recorded the water quality

3. Comment:

Maybe, add the discussion about the relationship between the environment with the fishes' conditions.

Respond:

The study showed that the fish are growing well during the dry season, this is related to environmental conditions during this season that the turbidity was lower and therefore the sunlight can penetrate into the waters maximumly and trigger the phytoplankton (algae) growing well. The algae are the primary food for mullets. We have mentioned this condition the pages 10-11.

4. Comment:

Many references were old references, need to add the new articles.

Respond:

Very limited reports on mullets were available, but we have added 4 related and up to date references (reference No. 45-48) to support the discussion

5. Comment:

The conclusion part is not enough.

Respond: 

We have extended the conclusion (on page 11)
